# Methodologies for the Analysis of HCV-Specific CD4^+^ T Cells

**DOI:** 10.3389/fimmu.2015.00057

**Published:** 2015-02-25

**Authors:** Megha U. Lokhande, Robert Thimme, Paul Klenerman, Nasser Semmo

**Affiliations:** ^1^Hepatology, Department of Clinical Research, University of Bern, Bern, Switzerland; ^2^Department of Gastroenterology and Hepatology, University Hospital of Freiburg, Freiburg, Germany; ^3^NIHR Biomedical Research Centre, Oxford and Peter Medawar Building for Pathogen Research, University of Oxford, Oxford, UK; ^4^Department of Hepatology, University Clinic of Visceral Surgery and Medicine, Inselspital, Bern, Switzerland

**Keywords:** viral hepatitis, hepatitis C, CD4 T cells, CD154, ELISpot, FACS, tetramers

## Abstract

Virus-specific CD4^+^ T cells play a major role in viral infections, such as hepatitis C virus (HCV). Viral clearance is associated with vigorous and multi-specific CD4^+^ T-cell responses, while chronic infection has been shown to be associated with weak or absent T-cell responses. Most of these studies have used functional assays to analyze virus-specific CD4^+^ T-cell responses; however, these and other detection methods have various limitations. Therefore, the important question of whether virus-specific CD4^+^ T cells are completely absent or primarily impaired in specific effector functions during chronic infection, has yet to be analyzed in detail. A novel assay, in which virus-specific CD4^+^ T-cell frequencies can be determined by *de novo* CD154 (CD40 ligand) expression in response to viral antigens, can help to overcome some of the limitations of functional assays and restrictions of multimer-based methods. This and other current established methods for the detection of HCV-specific CD4^+^ T cells will be discussed in this review.

## Introduction

Hepatitis C virus (HCV) infection is a serious healthcare problem chronically affecting 170–200 million people worldwide (1), which is approximately 3% of the world’s population (2–4). Hepatotropic viruses, such as HCV, can lead to severe liver disease, such as liver cirrhosis and hepatocellular carcinoma (HCC) (5, 6). HCV is responsible for about 3–4 million infections per year and deaths of about 476,000 HCV-infected patients from HCV-associated diseases and their complications (4). Only around 30% of HCV-infected adults are able to clear the virus spontaneously and are often asymptomatic. Innate and adaptive host immune responses play an important role in eradication of the virus. No protective vaccine is yet available against HCV infection (7).

T cells are highly specific immune cells involved in adaptive immune responses. Through their antigen-specific T-cell receptor (TCR), T cells identify antigens specifically, as well as efficiently; these expand into specific effector responses, with a broad repertoire of functions, and eventually contract, forming a memory response. As important effector cells in the defense against pathogens such as HCV, T cells are likely the most highly scrutinized cell type in the immune system.

The cellular components of the adaptive immune response, i.e., CD4^+^ helper and CD8^+^ cytotoxic T-cell-mediated immune responses, have been shown to play a central role in determining the outcome of HCV infection (8). Spontaneous viral clearance of HCV infection is characterized by early, strong, vigorous, polyclonal, and multi-specific T-cell responses during the acute phase of infection (9, 10); whereas, chronic HCV infection is associated with late, transient, weak, or narrowly focused specific T-cell responses (11–13). These data, along with strong associations between HLA Class I and II genes in outcome (14–16), point to the involvement of T cells in controlling the infection. In addition, during persistence of HCV infection, typically only low frequencies of HCV-specific T cells are reported in blood, although with a potential higher frequency in the liver (17, 18), which is the primary site of infection. However, these are functionally weak T-cell responses leading to the development and maintenance of chronic HCV infection (11, 19, 20).

However, the data on the function and specificity of T cells in chronic HCV remain quite limited, partly due to methodological constraints. Given their importance in defining both disease outcome and potentially the progression of pathology, further information about the frequencies, phenotypes, and functional capacities of HCV-specific T-cell immune response would be of value.

Traditionally, the main effector cells that eradicate HCV-infected cells were considered to be the cytotoxic T lymphocytes (CTLs) (21). In terms of CD4^+^ T helper cell responses, much attention has been focused on type 1 or “Th1” CD4^+^ T cells, since secretion of interferon-γ has been proposed to be linked to control of hepatotropic viruses (22). CD4^+^ T cells are central to the adaptive immune response to potentially act in different ways to initiate and maintain adaptive immunity, such as providing help for CD8^+^ T cells by cytokine production and activation of antigen-presenting cells (APCs) and many other mechanisms. During HCV infection, CD4^+^ T-cell responses are observed to be very different in chronic and resolved individuals; Why CD4^+^ T-cell responses may fail in acute infection leading to chronic infection is a critical and unanswered question in the field. Therefore, it is important to develop specific and sensitive detection methods for HCV-specific CD4^+^ T cells.

## Difficulties in Assessing HCV-Specific CD4^+^ T Cells

The frequency of T cells specific for a single peptide–MHC ligand is very low in naive repertoires, (range 0.2–60 cells/10^6^ naive T cells) due to the high diversity of T cell repertoire, allowing a response to a different variety of antigens (23, 24). In the memory repertoire and in the absence of acute infections, the frequency of specific T cells in peripheral blood is typically well below 1% (23). Therefore, the major issue for the detection and identification of antigen-specific T cells is the detection of rare events. Specifically, pathogen-specific CD4^+^ T cells are often circulating in low frequencies in un-manipulated samples, i.e., less than 0.01–0.1%, and such antigen-specific CD4^+^ T cells are 10- to 100-fold less frequent than cytotoxic T cells (25). The frequency of pathogen-specific T cells can vary widely depending on the nature of the pathogen, the status of the immune response, and the persistence or clearance of the pathogen. Interestingly, the functionally important T-cell populations may occur at even lower frequencies, and such populations may require additional enrichment for detection. This includes the *in vitro* expansion of antigen-specific CD4^+^ T cells, magnetic enrichment to collect infrequent target cells from large cell samples, enrichment of cytokine-secreting cells, and tetramer enrichment techniques. Therefore, highly specific labeling methods, which are capable of processing large cell samples to detect rare specific T cells within the large numbers of non-specific cells are necessary and several methods have been proposed to assess HCV-specific CD4^+^ T cells.

## Methods and their Limitations

**Figure 1:**
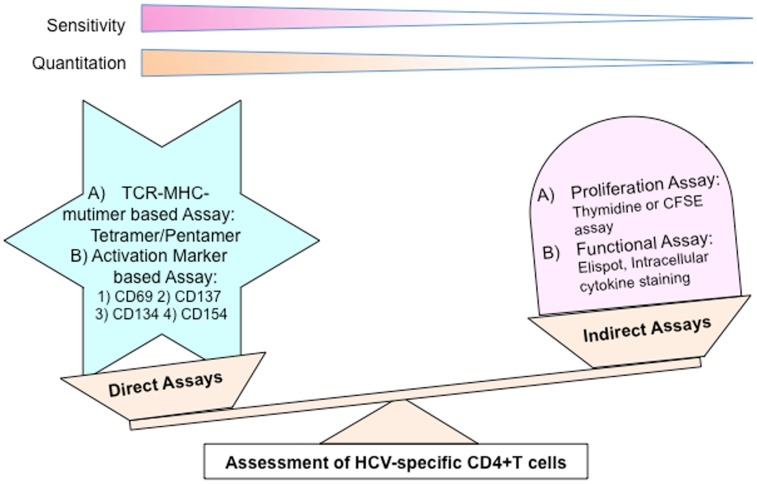
**Different methods to analyze HCV-specific CD4+ T cells**.

Several methods have been utilized to analyze HCV-specific T cells and can be divided into two groups (Figure 1): (A) indirect assays; (B) direct assays.

### Indirect assays

These assays depend on the functional characteristics of antigen-specific T cells after particular triggering of TCR, such as proliferation capacity, cytokine secretion, etc. Lefkovits et al. first described limiting dilution assay (26), by which the frequencies of antigen-specific CD4^+^ T cells participating in an immune response after particular stimulation were assessed (27) with estimations ranging from 1 in 10,000 to 1 in 1,000,000 PBMC. Traditionally, besides being extremely time consuming, it was technically difficult to identify rare cells of interest at frequencies below 10^−3^–10^−4^, thus making it hard to obtain reliable results. With the emergence of the latest high speed techniques, such as analyzers and sorters, these limitations could be overcome.

#### Proliferation assay

Thymidine incorporation assay is an assay that has been used for decades to measure the low frequencies of antigen-specific T cells on the basis of antigen-specific proliferation directly. In this assay, a radioactive nucleoside, ^3^H-thymidine, is incorporated into new strands of chromosomal DNA during mitotic cell division. It is measured by scintillation Beta counter in terms of radioactivity in DNA recovered from the cells in order to determine the extent of cell division due to the specific stimulation. This method can massively alter the phenotypic and functional properties of reactive cells and cell viability. However, the method has several limitations, including lower sensitivity, background DNA synthesis in other cells, and bystander cell activation. It was observed that PBMC proliferation cannot be equated with CD4^+^ T-cell proliferation because B cells and CD8^+^ T cells have also been shown to proliferate in response to recombinant viral proteins and/or their breakdown products (28). In a chimpanzee model study, peripheral HCV-specific CD4^+^ T-cell responses were observed in all HCV-infected animals without any correlation to the outcome of infection and independent of the kinetics, strength, specificity, or diversity of that response. However, a strong correlation between the intrahepatic HCV-specific T-cell response and course of infection could be found (29).

**Figure 2:**
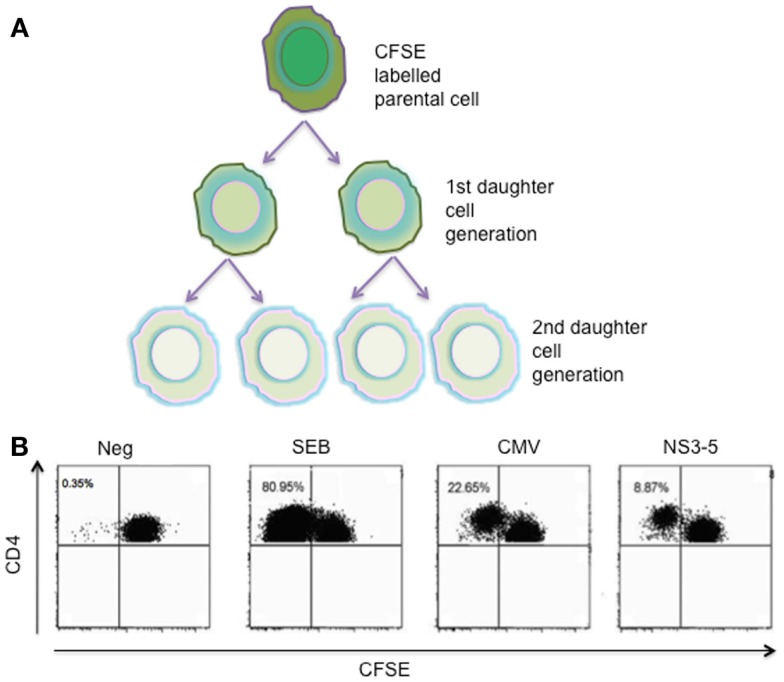
**CFSE proliferation assay**. **(A)** Decreasing fluorescence by equal distribution after each cell division. **(B)** Representative CFSE FACS plots: CFSE-labeled PBMCs on day 6 following stimulation with SEB or PHA, CMV, and HCV NS3–5 proteins are shown. Undivided CD4^+^ T cells are detected in the upper right quadrants of each FACS plot, and the CFSE signal is diluted with each cell division as the dye is distributed to the daughter cells. Numbers in the upper left quadrants of each plot represent the percentage of antigen-specific CD4^+^ T cells that have proliferated during the 6-day culture. SEB, staphylococcal enterotoxin B; PHA, Phytohemagglutinine; CMV, cytomegalovirus; CFSE, carboxyfluorescein diacetate succinimidyl ester; NS3-5, pool of HCV non-structural proteins 3, 4 and 5.

CFSE staining, a flow-cytometric approach, is used to directly monitor the rate of lymphocyte proliferation, due to progressive halving of CFSE fluorescence in cells following cell division (30) (Figure 2). Technically, CFSE can be toxic to cells at high concentrations, and it is therefore necessary to determine the optimum labeling conditions that give good fluorescence and preserve normal function. Furthermore, the analysis of dead cells by apoptosis during the time of analysis is not possible with this method. Therefore, the evaluation made with the technique, in which 3–5 days *in vitro* culture period is necessary, is not a direct evaluation of T cells dividing in response to specific antigen stimuli. In the case of HCV infection, the proliferative capacity assessed by the dilution of CFSE was analyzed in our previous study, in which only a minority of chronic HCV cases demonstrated HCV-specific proliferation with highest frequency of 0.46% proliferating CD4^+^ T cells. In contrast, strong proliferative responses were found in resolved HCV individuals (31). In conclusion, the lack of proliferative capacity of CD4^+^ T cells is linked to persistent HCV-infected cases.

Although there are technical limitations for these assays, HCV-specific CD4^+^ T cells are mostly non-proliferative in such assay systems; thus, for studies of CD4^+^ T-cell responses during chronicity, these approaches are very limited.

#### Other functional assays

**Figure 3:**
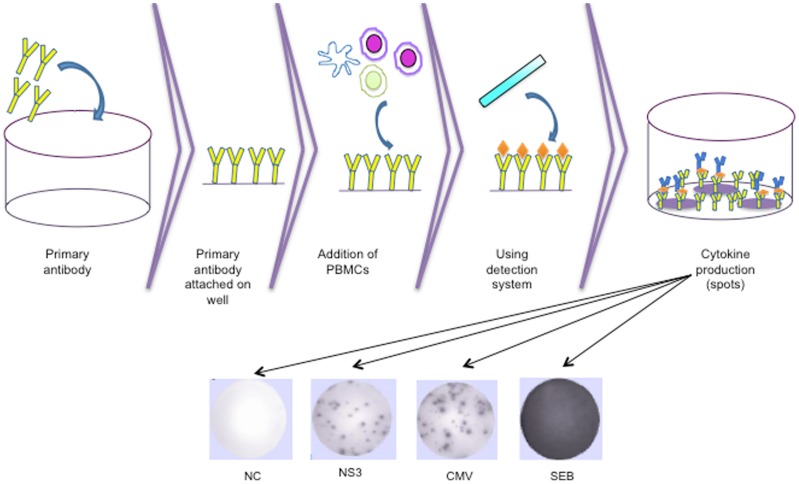
**Diagram of the technical procedure of T-cell ELISpot and representative ELISpot wells for a negative control, NS3, CMV lysate, and SEB, respectively**. CMV, cytomegalovirus; SEB, staphylococcal enterotoxin B.

ELISpot is an established method for characterizing the T cell response in which magnitude and quality of T-cell immunity is measured at single cell resolution by detecting individual events of antigen-specific T cells that engage in secretion of cytokines, such as IFN-γ, IL-2, etc. (Figure 3). In the case of HCV infection, previous studies revealed that HCV-specific CD4^+^ T cells were often unable to produce cytokine after stimulation with HCV antigen in chronic HCV patients, whereas, strong CD4^+^ T-cell IFN-γ responses were observed in resolved HCV patients (19, 31–33). Correspondingly, IL-2 secretion capacity was also similar to IFN-γ secretion capacity in chronic HCV-infected cases (31, 32). In contrast, in another study, cytokine secretion could be detected in chronic HCV at least for core peptides; however, this was not the case for the non-structural regions (19). Overall, due to relative dysfunctionality of these cells in chronic HCV infection, especially for those directed against non-structural proteins, this method does not provide complete information about the actual frequency of antigen-specific T cells.

In flow cytometry cytokine production assays, TCR-activated T cells produce cytokines transiently. Different cytokines have different kinetics in the response of specific antigen. Intracellular cytokine production can be detected using Brefeldin A and Monensin, which interfere in protein-trafficking events. In addition, cytokines can be detected on the cell surface of the secreting cells by retention of the secreted cytokine via a cell-surface affinity matrix (34, 35). Therefore, methods based on the detection of HCV antigen-reactive cytokine expression are independent of proliferative capacity, MHC alleles, and peptides. However, HCV-specific T cells that do not produce cytokines after activation (31) or which produce alternative cytokines not in the assay protocol (e.g., type 2 or type 17) may be missed. Furthermore, use of one defined cytokine for detection of CD4^+^ T cells may lead to biased evaluation of the actual frequencies of HCV-specific CD4^+^ T cells.

### Direct methods

In these methods, detection depends on direct labeling of antigen-specific T cells with fluorescent molecules, such as MHC Class II–peptide multimers (tetramers/pentamers) or particular receptor antibodies, and can be analyzed by flow cytometry.

With the traditional methods based on function, the detection of the actual frequency and phenotype of reactive T cells, as well as elimination of bystander proliferation, were difficult to avoid. Therefore, to achieve accuracy, flow cytometry techniques have advantages, and also provide an opportunity to examine multiple parameters of single cells from large cell samples. Polychromatic cytometry for identification of rare CD4^+^ T cells has the capacity to gain maximum information, e.g., up to 20 parameters from a single analysis. Limitation of flow-cytometric assays to detect rare events can further be minimized by a pre-enrichment strategy with magnetic cell separation, which collects the small number of rare events from large cell numbers (34).

#### Labeling with peptide–MHC-multimers

**Figure 4:**
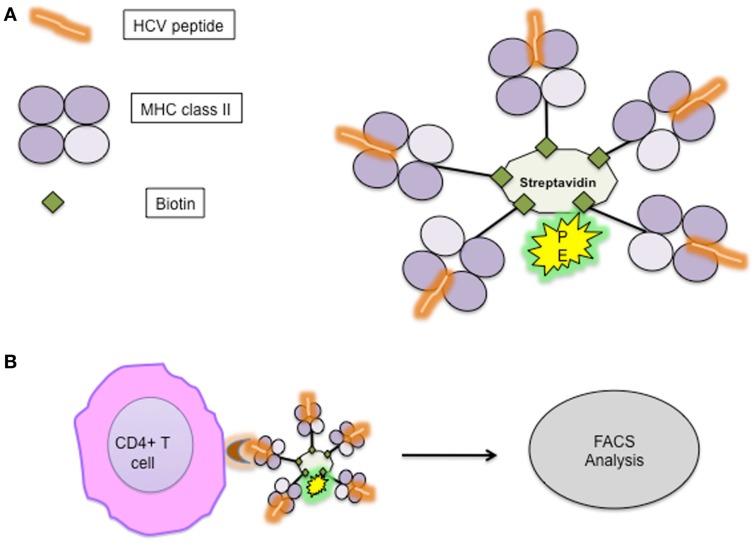
**(A)** Outline of MHC-pentamer complex with HCV-specific peptide; **(B)** specific CD4^+^ T cells bind with HCV-specific pentamer complex. MHC, major histocompatibility complex.

Detection of antigen-specific CD4^+^ T cells is possible *ex vivo*, based on direct labeling with specific peptide–MHC-multimers without restriction to certain functional parameters (Figure 4). The low binding affinity of TCR to MHC–peptide monomers (36) was overcome by multimerization of peptide–MHC complexes (37). Initially, MHC class II multimers were difficult to construct due to the problems of yield, the variety of MHC class structure, and peptide affinity; recently, however, different varieties of MHC II multimers for specific recognition of CD4^+^ T cells have become commercially available.

However, there are some limitations for the tetramer technology, such as the knowledge of immunodominant epitopes and exact characterization of the MHC alleles of the patients are required. Hence, the detection of the entire repertoire of antigen-specific CD4^+^ T cells appears difficult (25). Additionally, there still remain issues regarding frequencies, since when using non-specific MHC II–peptide tetramers, background staining may approach 0.1% (38).

#### Labeling with activation markers

Prior specific stimulation with cognate antigen, i.e., single peptides, proteins, or whole antigen lysates, is necessary for detection of antigen-specific T cells on the basis of functional parameters. T-cell antigen reactivity-based assays to enumerate antigen-specific T cells have the advantage that they are independent of MHC alleles and not restricted to single peptides.

Independent of functional parameters, such as cytokine secretion or cytotoxicity, another approach for accessing the antigen-specific T cells by flow cytometer is on the basis of activation markers. The transient expression of activation markers on the T-cell surface depends on antigen-specific activation by TCR triggering. These activation markers may be constrained to a differentiation state of the T cells (e.g., naïve, central, effector memory) or T cell types. Several potential activation markers have been proposed, such as CD69, CD25, CD71, HLA-DR, CD134 (OX40), CRTAM, CD137 (4-1BB), and CD154 (CD40-L) (23). However, some limitations for many of these markers restrict precise enumeration due to sensitivity to bystander activation (CD69, CD25), constitutive expression on specialized T cell subsets (CD69, CD25, CRTAM), or late up-regulation after stimulation (HLA-DR, CD134, CD71) (23).

##### CD69

The extensively used activation marker, CD69 is expressed on activated CD4^+^ and CD8^+^ T cells, B cells, or NK cells. Nevertheless, non-stimulated T cells restrict the accurate enumeration of antigen-specific T cells (39). There is evidence that CD69 expression is not exclusively dependent on TCR activation (40).

##### CD137

Expression of CD137 (4-1BB), a member of the TNFR superfamily, is observed on CD4^+^ and CD8^+^ T cells after specific stimulation (41–43) and even on CD4^+^ Foxp^+^ regulatory T cells (44). Hence, CD137 expression-based assay can be used to detect antigen-specific CD4^+^ T cells after TCR activation with specific stimulation.

##### OX40

Similarly, recently a novel assay system has been developed and validated for the detection of HCV-specific CD4+ T cells. The assay system is based on *ex vivo* stimulation with HCV antigens, and HCV-specific CD4^+^ T cells can be detected with flow cytometry after staining with CD25 (IL-2R α) and CD134 (OX40) (45). TCR triggering stimulates up-regulation of CD25 and CD134 over 24–48 h, with the optimal readout determined to be 44 h (46). It is a highly sensitive method, which correlates with CFSE-based LPA, and successfully detects HCV-specific CD4^+^ T-cell responses in resolved and chronic HCV-infected patients (45), as well as HIV-infected individuals (47).

## CD154 (CD40-L) Assay

This recently established method analyzing expression of CD154 is highly sensitive and specific for the overall assessment of antigen-specific CD4^+^ T cells. It avoids many of the limitations described above for the virus-specific CD4^+^ T cell detection. Hence, a virus-specific CD4^+^ T cell response can be detected not only through an antiviral function of CD4^+^ T cells, but also staining of PBMCs with surface marker CD154 (CD40-L) (31) or intracellular CD154 expression after stimulation with cognate antigen (48).

**Figure 5:**
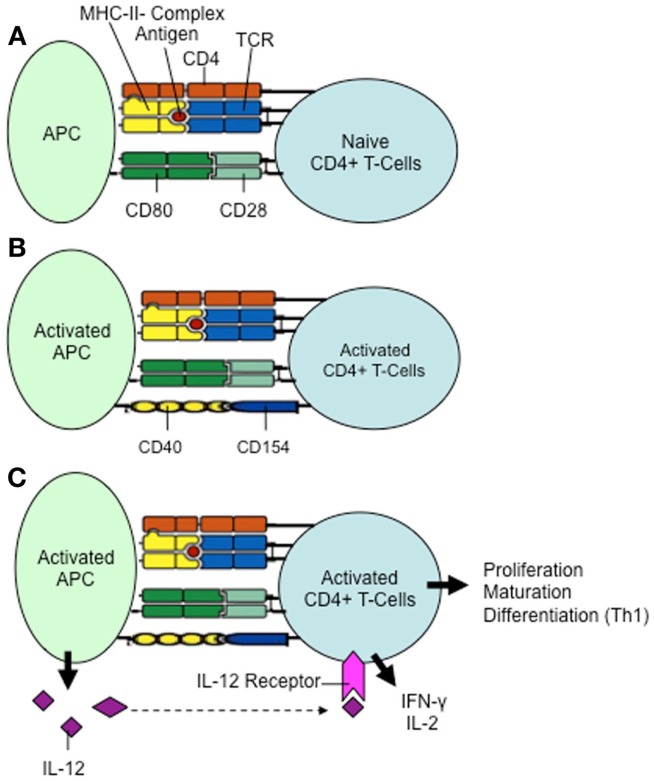
**The role of CD154 in the activation of CD4^+^ T cells**. **(A)** Activation of naive CD4^+^ T cells requires two signals: (1) binding of the TCR (T cell receptor) to the antigen-loaded MHC II complex on the antigen-presenting cell (APC). (2) Interaction of the co-stimulatory molecules CD28 and CD80. **(B)** Activated antigen-specific CD4^+^ T cells express CD154, which binds to the CD40 molecule on the surface of the antigen-presenting cell. Simultaneously, the further differentiation of the antigen-presenting cell is initiated via CD40/CD154-signaling, in which more co-stimulatory molecules are expressed (not shown). **(C)** The fully activated antigen-presenting cell now increasingly secretes IL-12. Proliferation, maturation, and differentiation of CD4^+^ T cells into Th1 lymphocytes with secretion of IFN-γ and IL-2 can be induced.

CD154, a type II member protein of 33 kDa, which belongs to the family of tumor necrosis factors (TNF), also called CD40 ligand (CD40-L), is a cell-surface molecule present primarily on activated T cells. Engagement of its receptor, CD40, on APCs results in priming and expansion of antigen-specific CD4^+^ T cells, induction of co-stimulatory molecules on APCs, and the release of cytokines (49) (Figure 5). This molecule plays a key role in the activation of antigen-specific CD4^+^ T cells; this is evident by treatment of blocking antibodies against CD154, leading to an activation inhibition of antigen-specific CD4^+^ T cells (50) (Figure 5).

In addition to the detection of live antigen-specific CD4^+^ T cells, it is independent of other effector functions, such as proliferation or cytokine production, and independent of MHC haplotype or immunodominant epitopes. The method allows the analysis of co-expression of CD154 and effector cytokine production, such as IFN-γ or IL-2 and/or the phenotypes of the T cells with other surface markers. The detection of CD154 expression for assessing viral-specific CD4^+^ T cells is therefore a method which permits a quantitative and qualitative *ex vivo* and *in vitro* evaluation of antigen-specific CD4^+^ T cells. The CD154 expression-based method for the analysis of antigen-specific CD4^+^ T cells allows the identification of activated T cells even when their capacity to secrete cytokines is inadequate, such as in chronic HCV-infected individuals. The method is suitable for whole blood analysis (51).

In chronic HCV infection, one of the main reasons for the difficulty in assessing antigen-specific CD4^+^ T cells is their low frequency. Day et al. used MHC tetramer to detect antigen-specific CD4^+^ T cells and estimated about 1:1200–1: 111,000 frequencies of those cells (52). Interestingly, Möller et al. detected an average of 100,000–150,000 CD4^+^ T cells in analysis in the method based on CD154 expression (53). They used threshold in analysis around 0.01%; therefore, only T-cell populations with a frequency above 1:10,000 could be detected.

## Lessons from Studying CD154 Expression in HCV Infection

**Figure 6:**
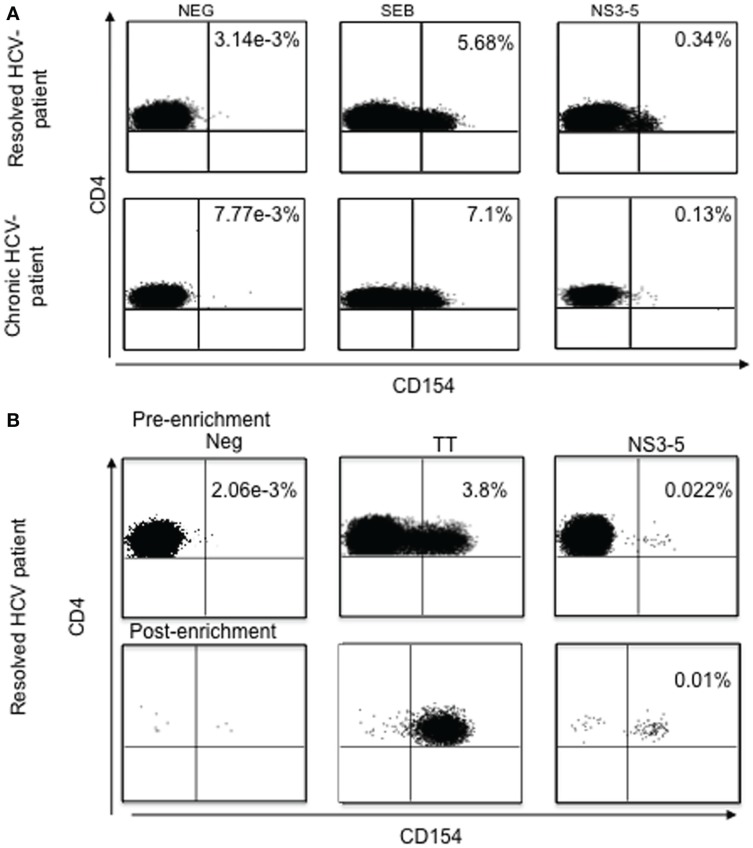
**(A)** *Ex vivo* analysis of antigen-specific CD154 expression without enrichment. Analysis of antigen-specific CD154+ CD4+ T cells from two representative individuals with spontaneously resolved infection and chronic HCV infection, respectively. SEB, staphylococcal enterotoxin B. **(B)** *Ex vivo* analysis of antigen-specific CD154 expression pre- and post-enrichment using the magnetic bead enrichment assay. TT, tetanus toxoid; NS3-5, pool of HCV non-structural proteins 3, 4 and 5.

In recent studies, CD154 expression was examined on CD4^+^ T cells after stimulation with HCV-cognate antigen in peripheral blood from HCV chronic infected patients and in spontaneous viral resolved (SVR) HCV patients (31) (Figure 6). The results of these studies demonstrated that HCV-specific CD4^+^ T cells were present not only in spontaneous resolved HCV, but also in chronically infected HCV individuals. Similar findings by Bes et al. proved that HCV-specific CD4^+^ T cells, although dysfunctional, are present in the peripheral blood in most patients with chronic HCV infection and can be easily detected, irrespective of their functional profile, by transient antigen-specific up-regulation of CD154 (54).

Indeed, in our previous findings, detection of CD154 expression could be found in chronic HCV-infected patients, with frequencies of HCV-specific CD4^+^ T cells almost comparable with spontaneous viral clearance. However, when these frequencies were assessed for functional capacity, IFN-γ and IL-2 secretion, as well as proliferation, were significantly lower in chronic HCV infection when compared with the spontaneous HCV-resolved individuals, suggesting that HCV-specific CD4+ T cell responses are present in chronic HCV, although in a dysfunctional state (31). Several studies utilizing MHC class II tetramers have supported the conclusion that CD4^+^ T-cell responses in the majority of chronically infected HCV patients are absent (52). However, CD154 expression-based analysis of HCV-specific CD4^+^ T cells indicates that the HCV-specific CD4^+^ T cells are present in the peripheral blood and even liver, but they are dysfunctional. Several studies suggested that HCV-specific CD4^+^ T cells might be sequestered at the site of viral replication and inflammation (31, 55–58), and the CD154 up-regulation studies found higher antigen-specific CD4^+^ T-cell frequencies in the liver than the blood in HCV-infected patients. Overall, these studies indicated that the virus-specific CD4^+^ T cells are present and even enriched at the site of disease, and that the virus can persist despite the presence of these virus-specific T cells.

One advantage of the CD154 method for the assessment of viral-specific CD4^+^ T cells is that responses can not only be detected using whole protein as antigen, but also using peptides, allowing fine mapping of epitopes (31). Thus, overall this is a simple method for reliably characterizing the targeted peptides after stimulation with corresponding antigens.

## Summary

In summary, antigen-specific CD4^+^ T-cell responses can be readily analyzed by detection of CD154 expression in HCV infection. This method is simple, reliable, and independent of both HLA type, knowledge of the epitopes, and effector functions, such as cytokine production or proliferation capacity of CD4^+^ T cells. Studies using CD154 expression-based methods for analysis of HCV-specific CD4^+^ T cells suggest that CD4^+^ T cells are not fully exhausted or deleted during chronic HCV infection, but some remain detectable and can trigger through their TCR. However, these cells typically lack proliferative capacity and cytokine secretion capacities, such as for IFN-γ and IL-2.

It will be of interest in the future to explore the mechanisms that lead to the dysfunction of virus-specific CD4^+^ T cells. The method to detect these “dysfunctional” antigen-specific CD4^+^ T cells in blood and at the site of infection, provides a new opportunity to pursue this important question experimentally and to understand the overall mechanisms that lead to chronic HCV infection. Defining the exact state of differentiation of CD154^+^ CD4^+^ T cells, the differences between chronically infected patients and patients with spontaneous viral clearance, and the impact of direct acting antivirals (DAA) therapy are all relevant questions for future study.

## Conflict of Interest Statement

The authors declare that the research was conducted in the absence of any commercial or financial relationships that could be construed as a potential conflict of interest.
